# A cluster-randomised, non-inferiority trial of the impact of a two-dose compared to three-dose schedule of pneumococcal conjugate vaccination in rural Gambia: the PVS trial

**DOI:** 10.1186/s13063-021-05964-5

**Published:** 2022-01-24

**Authors:** Grant A. Mackenzie, Isaac Osei, Rasheed Salaudeen, Ilias Hossain, Benjamin Young, Ousman Secka, Umberto D’Alessandro, Arto A. Palmu, Jukka Jokinen, Jason Hinds, Stefan Flasche, Kim Mulholland, Cattram Nguyen, Brian Greenwood

**Affiliations:** 1 Medical Research Council Unit The Gambia at London School of Hygiene & Tropical Medicine, Fajara, Gambia; 2 Faculty of Infectious & Tropical Diseases, London School of Hygiene & Tropical Medicine, London, UK; 3 Murdoch Children’s Research Institute, Melbourne, Australia; 4 Department of Paediatrics, University of Melbourne, Melbourne, Australia; 5 Finnish Institute for Health and Welfare, Helsinki, Finland; 6 Institute for Infection and Immunity St George’s University of London, London, UK; 7 BUGS Bioscience, London Bioscience Innovation Centre, London, UK; 8 Faculty of Epidemiology and Public Health, London School of Hygiene & Tropical Medicine, London, UK

**Keywords:** Cluster-randomised trial, Pneumococcal, Vaccine, Schedule, Carriage, Impact

## Abstract

**Background:**

Pneumococcal conjugate vaccines (PCV) effectively prevent pneumococcal disease but the global impact of pneumococcal vaccination is hampered by the cost of PCV. The relevance and feasibility of trials of reduced dose schedules is greatest in middle- and low-income countries, such as The Gambia, where PCV has been introduced with good disease control but where transmission of vaccine-type pneumococci persists. We are conducting a large cluster-randomised, non-inferiority, field trial of an alternative reduced dose schedule of PCV compared to the standard schedule, the PVS trial.

**Methods:**

PVS is a prospective, cluster-randomised, non-inferiority, real-world field trial of an alternative schedule of one dose of PCV scheduled at age 6 weeks with a booster dose at age 9 months (i.e. the alternative ‘1 + 1’ schedule) compared to the standard schedule of three primary doses scheduled at 6, 10, and 14 weeks of age (i.e. the standard ‘3 + 0’ schedule). The intervention will be delivered for 4 years. The primary endpoint is the population-level prevalence of nasopharyngeal vaccine-type pneumococcal carriage in children aged 2 weeks to 59 months with clinical pneumonia in year 4 of the trial. Participants and field staff are not masked to group allocation while measurement of the laboratory endpoint will be masked. Sixty-eight geographic population clusters have been randomly allocated, in a 1:1 ratio, to each schedule and all resident infants are eligible for enrolment. All resident children less than 5 years of age are under continuous surveillance for clinical safety endpoints measured at 11 health facilities; invasive pneumococcal disease, radiological pneumonia, clinical pneumonia, and hospitalisations. Secondary endpoints include the population-level prevalence of nasopharyngeal vaccine-type pneumococcal carriage in years 2 and 4 and vaccine-type carriage prevalence in unimmunised infants aged 6–12 weeks in year 4. The trial includes components of mathematical modelling, health economics, and health systems research.

**Discussion:**

Analysis will account for potential non-independence of measurements by cluster, comparing the population-level impact of the two schedules with interpretation at the individual level. The non-inferiority margin is informed by the ‘acceptable loss of effect’ of the alternative compared to the standard schedule. The secondary endpoints will provide substantial evidence to support the interpretation of the primary endpoint. PVS will evaluate the effect of transition from a standard 3+ 0 schedule to an alternative 1 + 1 schedule in a setting of high pneumococcal transmission. The results of PVS will inform global decision-making concerning the use of reduced-dose PCV schedules.

**Trial registration:**

International Standard Randomised Controlled Trial Number 15056916. Registered on 15 November 2018.

**Supplementary Information:**

The online version contains supplementary material available at 10.1186/s13063-021-05964-5.

## Administrative information

Note: the numbers in curly brackets in this protocol refer to SPIRIT checklist item numbers [1]. The order of the items has been modified to group similar items (see http://www.equator-network.org/reporting-guidelines/spirit-2013-statement-defining-standard-protocol-items-for-clinical-trials/).

**Table:** 

Title {1}	A cluster-randomised, non-inferiority trial of the impact of a two-dose compared to three-dose schedule of pneumococcal conjugate vaccination in rural Gambia
**Trial registration** **{2a} and {2b}**	International Standard Randomised Controlled Trials Number – 15056916. https://www.isrctn.com/ISRCTN15056916. Registered on 15 November 2018.
**Protocol version {3}**	Protocol version 4.0, 16 November 2020
**Funding {4}**	Bill & Melinda Gates Foundation (OPP1138798; INV006724); Medical Research Council (UK), Wellcome, UKAID, National Institute of Health Research (UK), Joint Global Health Trials scheme (MR/R006121/1); Medical Research Council Unit The Gambia at London School of Hygiene & Tropical Medicine
**Author details {5a}**	Grant A Mackenzie^1,2,3,4^, Isaac Osei^1,2^, Rasheed Salaudeen^1^, Ilias Hossain^1^, Benjamin Young^1^, Ousman Secka^1^, Umberto D’Alessandro^1,2^, Arto A Palmu^5^, Jukka Jokinen^5^, Jason Hinds^6,7^, Stefan Flasche^8^, Kim Mulholland^3,4,8^, Cattram Nguyen^3^, Brian Greenwood^2^ 1 Medical Research Council Unit The Gambia at London School of Hygiene & Tropical Medicine, Fajara, The Gambia. 2 Faculty of Infectious & Tropical Diseases, London School of Hygiene & Tropical Medicine, London, UK. 3 Murdoch Children’s Research Institute, Melbourne, Australia. 4 Department of Paediatrics, University of Melbourne, Australia. 5 Finnish Institute for Health and Welfare, Finland 6 Institute for Infection and Immunity, St George’s, University of London, London, UK 7 BUGS Bioscience, London Bioscience Innovation Centre, London, UK 8 Faculty of Epidemiology and Public Health, London School of Hygiene & Tropical Medicine, London, UK.
**Contact information for trial sponsor {5b}**	London School of Hygiene & Tropical Medicine, Keppel Street, London, WC1E 7HT, UK. Contact name: Head of Research Governance and Integrity, RGIO@lshtm.ac.uk.
**Role of sponsor and funder {5c}**	The trial sponsor is not involved in the study design; collection, management, analysis and interpretation of data; writing of the report; the decision to submit the report for publication, and will not have authority over any of these activities. The funders contributed to the study design but are not involved in the collection, management, analysis and interpretation of data; writing of the report; the decision to submit the report for publication, and will not have authority over any of these activities.

## Introduction

### Background and rationale {6a}

Despite the pneumococcus causing more childhood deaths than any single pathogen [2, 3], global control of pneumococcal disease is hampered by the cost of pneumococcal conjugate vaccines (PCVs). In addition to the relatively high cost of several new vaccines that have recently been introduced in many low-income countries, expanded programmes on immunisation (EPI) face the additional challenge of schedules with increasing numbers of doses. Reducing the cost and complexity of EPI schedules would improve the flexibility, acceptability and sustainability of immunisation programmes.

Low-income countries receive subsidised PCV through the GAVI Alliance, providing a co-payment of 0.15–0.30 USD per dose (increasing 15% per year in ‘intermediate’ countries) [4]. However, when countries’ Gross National Income per capita exceeds the World Bank ‘low-income’ threshold of ~ 1500 USD, they begin to transition from GAVI support. During transition, co-payments increase each year for 5 years to a final price set under the GAVI Advance Market Commitment (2.00–2.90 USD per dose) [5]. GAVI expenditure on PCV represents approximately half of its vaccine budget [6]. The importance of the cost of PCV was evident in The Gambia where its introduction, at 0.2 USD per dose, increased the national cost of the EPI programme by one third, with vaccine representing 91% of the total cost of introducing PCV [7]. Thus, a major determinant of the sustainability of pneumococcal vaccination in low- and middle-income countries is vaccine cost. Middle-income countries, ineligible for GAVI support, experience many child deaths due to pneumococcus but cost has precluded many from introducing PCV.

EPI programmes in low- and middle-income countries are becoming more complicated and costly with the introduction of new vaccines. The addition of vaccines such as PCV, rotavirus vaccine, injectable polio vaccine, meningococcal group A conjugate, human papillomavirus vaccine, and typhoid conjugate challenges the implementation, acceptance, cold-chain capacity, and sustainability of EPIs. The difficulty of introducing such new vaccines has its biggest impact in low- and middle-income countries where the burden of disease is greatest but resources are scarce.

The Medical Research Council Unit The Gambia at London School of Hygiene & Tropical Medicine (MRCG at LSHTM) has a long history investigating the burden of pneumococcal disease and pneumococcal vaccination. In 2000–2004, a trial of a 9-valent PCV (PCV9) was conducted in Central and Upper River Regions (CRR and URR) of The Gambia. Vaccine efficacy in children aged 3–29 months was 37% against radiological pneumonia, 77% against vaccine-type (VT) invasive pneumococcal disease (IPD), and 16% against all-cause mortality [8]. In 2009, The Gambia introduced PCV7 into the routine EPI using a three-dose schedule without a booster dose (i.e. a ‘3 + 0’ schedule). In 2011, PCV7 was replaced by PCV13. The Pneumococcal Surveillance Project (PSP) has documented the impact of PCV13 in the Basse Health & Demographic Surveillance System (BHDSS) in rural Gambia. Four to five years after the introduction of PCV7/13 the incidence of VT IPD had declined by 82%, with a 24% reduction in radiological pneumonia, and 61% reduction in severe hypoxic pneumonia in children aged 2–59 months [9, 10]. Eight years after the introduction of PCV7/13 the incidence of VT IPD in the 2–59 month age group has declined by 92% and radiological pneumonia has declined by 33% [11]. Before the introduction of PCV, PSP detected an average of 35 annual cases of VT IPD among children aged 2–59 months. In 2016, we detected six cases of VT IPD, and in 2017, we detected three. In 2016/2017, we detected zero cases of VT IPD among children in the first year of life. These data indicate that the introduction of PCV7/13 has now controlled VT IPD.

It is now evident that following the introduction of PCV13, herd protection has developed in The Gambia. The annual count of VT IPD in older children in PSP was six to ten before the introduction of PCV13 in 2011. Following the introduction of PCV13 the annual case counts in 2015, 2016, and 2017 were four, one, and zero, respectively. In the 5–14 year age group, IPD incidence declined by 69% (95%CI, − 28–91%) and radiological pneumonia by 27% (95%CI, − 5–49%) [11]. Similar findings of the direct and herd effect of PCV are evident in Kenya [12, 13].

The prevalence of nasopharyngeal (NP) carriage of PCV13 VT in the BHDSS area before the introduction of vaccine was 47% in the under-5-year age group. In 2015 and 2017, the prevalence of vaccine types was 15% and 17%, respectively (author’s own data). The downward trajectory of VT prevalence from 2009 to 2015 and 2017 suggests that the introduction of PCV13 has substantially reduced the prevalence of VT carriage. However, it is evident that transmission of VT pneumococci continues in the population. Ongoing circulation of VT pneumococci following the introduction of PCV is also observed in Kenya with a persisting 9% prevalence in young children [12], and particularly so in Malawi with 17% prevalence in 3–5-year-olds [14, 15]. Kenya and Malawi, like The Gambia, have high rates of pneumococcal transmission and all three countries use the standard 3 + 0 schedule.

Mathematical models can help to understand pneumococcal transmission under different vaccination scenarios. Such models may benefit from inputs on population contact patterns. Evidence suggests that interpersonal physical contact frequency is associated with pneumococcal carriage and that older unvaccinated children and larger household sizes, typical in low-income countries, may drive pneumococcal transmission [16]. Children aged 1–5 years are large contributors to pneumococcal transmission from increased physical contacts and high rates of carriage [17]. Interpersonal contact patterns and their relationship to pneumococcal carriage will be important inputs into modelling the impact of reduced dose schedules of PCV in high transmission settings.

The Gambian EPI schedule currently includes birth doses of BCG, hepatitis B and oral polio vaccine (OPV); visits at 2 and 3 months of age when OPV, pentavalent, rotavirus and PCV13 vaccines are scheduled; a visit at 4 months of age when OPV, injectable polio (IPV), pentavalent, rotavirus and PCV13 vaccines are scheduled; a visit at 9 months of age when measles-rubella and yellow fever vaccines are scheduled; conjugate meningococcal group A vaccine scheduled at 12 months of age was introduced in 2018; at 18 months of age OPV and measles-rubella vaccines are scheduled. Human papilloma virus vaccine delivered to school-age girls was introduced in 2020. In the event that polio is eradicated then OPV will be phased out and be replaced by IPV. The EPI is also considering the introduction of a conjugate typhoid vaccine. Thus, in recent years, and in the anticipated future, the EPI schedule has introduced and may introduce additional injectable antigens.

Several studies indicate that immunological priming for an optimal PCV booster dose response may be more effective with fewer primary doses [18, 19]. In addition, the immunological response to a booster dose following a single priming dose may reduce VT acquisition to a greater degree than following the standard 3 + 0 schedule [20]. As a result, a schedule with one primary dose and a later booster dose, that is a 1 + 1 schedule, may induce greater herd protection than the 3+ 0 schedule. An accompanying paper describes the protocol for a trial in which we are investigating the immunogenicity of the comparator schedules, testing whether the concentration of VT serotype-specific IgG is greater in the 1 + 1 compared to the 3 + 0 group at 18 months of age.

This trial joins a global initiative to generate evidence concerning reduced dose schedules for PCV. WHO is engaged with this initiative having held a consultative meeting in February 2016. Studies investigating reduced dose schedules for PCV are underway in South Africa, Vietnam, India, and the UK. The UK introduced the 1 + 1 schedule nationwide in 2019 [21]. Our trial in The Gambia is critical to provide evidence from a typical African setting.

### Rationale {6a}

Given the control of VT IPD by routine immunisation, we aim to compare the community-level impact of an alternative PCV schedule (doses scheduled at ages 6 weeks and 9 months) with the standard schedule (doses scheduled at age 6, 10, and 14 weeks). The authors’ meta-analysis of observational studies found that one infant dose of PCV was around 65% effective against VT IPD, less than that of two or three doses (85% to 90%) [22–24]. The median age at vaccination in the analysed observational studies was greater than 2 months and so this analysis is likely to overestimate the efficacy of one dose given at 6 weeks of age. In the USA, the effectiveness against VT IPD of one dose given before the age of 7 months was 84% in the 6 months after vaccination but effectiveness then waned [22]. In Fiji and The Gambia, one dose of PCV at 2 or 3 months of age significantly reduced carriage of VT pneumococci at 9 months of age [19, 25]. A Dutch study showed that following two doses of PCV7 at the ages of 2 and 4 months, a booster dose at 11 months prevented VT carriage in the 2nd year of life [20].

Data from the UK trial show that the immunogenicity of the PCV booster dose using a 1+ 1 schedule was equivalent to, or superior to, a 2+ 1 schedule for nine of the 13 serotypes in PCV13 [18]. Of importance to the Gambian setting, where serotypes 1 and 14 have been the most common serotypes causing IPD, IgG responses to those serotypes following the booster dose were superior in the 1+ 1 group. Almost all infants in both the 1 + 1 and 2 + 1 groups had IgG responses above the protective titre of 0.35 μl/ml, for all serotypes except serotype 3, for which fewer reached protective thresholds in both schedules. Geometric mean IgG concentrations following the primary series were higher in the 2+ 1 schedule, although differential time from vaccination to sampling in the two groups biased results towards lower levels in the 1+ 1 group. There is also suggestive clinical evidence that herd protection following the use of a 2+ 1 schedule is greater than with a 3 + 0 schedule [26].

The duration of protection of PCV is poorly defined but the potential for greater antibody persistence following a booster dose compared to doses in early infancy [27, 28] suggests that protection may be more long-lived when a booster dose is given [29]. Even though PCV has proven efficacious against serotype 1 in young African children, serotype 1 continues to cause epidemic meningitis in the African meningitis belt [30]. There is a strong rationale to test, in our epidemiological setting, whether a 1 + 1 schedule will provide an overall non-inferior programmatic effect compared to the 3 + 0 schedule. It is important to note that as the time course after the introduction of a vaccine extends, the direct effect of vaccination becomes less important and herd protection assumes an increasingly important role [31].

Testing the non-inferiority of the 1+ 1 compared to 3+ 0 schedule will provide necessary evidence to assist decision-makers in considerations of reduced dose schedules. Reducing the number of PCV doses in EPI schedules will impact on multiple elements of the challenges posed by this vaccine; reducing the costs to countries and GAVI, reducing the number of injections in schedules and providing greater flexibility for the inclusion of other vaccines, reducing staff time and cold-chain requirements, and ultimately making EPI programmes more acceptable and sustainable. If countries can safely transition to 1 + 1 schedules the global uptake of PCV should accelerate with greater and more sustainable reductions in the pneumococcal disease.

### Objectives {7}

The objective of the PVS trial is to generate evidence to inform global and national policy concerning the use of an alternative PCV schedule with one priming and one booster dose compared to the standard schedule of three primary doses to control pneumococcal carriage and disease. Exploratory measures will inform the time course of development of the differential effects of the two schedules.

We hypothesise that following the control of VT IPD by routine immunisation with three doses of PCV13, the community-level impact of the alternative schedule (doses scheduled at ages 6 weeks and 9 months) will be non-inferior to the standard schedule (doses scheduled at age 6, 10, and 14 weeks). We hypothesise that the alternative schedule will provide adequate direct protection between 2 and 9 months of age, during which time the risk of VT disease in our setting is very low, and potentially superior herd protection, and similar overall effectiveness compared to the standard schedule. An accompanying paper describes the protocol for a related trial in which we are investigating whether the rate of acquisition of VT pneumococci is reduced in the 5 months after the administration of the booster dose, in the 1 + 1 compared to the 3 + 0 group.

### Trial design {8}

PVS is a parallel-group, phase IV, unmasked, cluster-randomised, non-inferiority field trial of the population-level impact of an alternative compared to the standard schedule of PCV13. We will test the non-inferiority of the impact of the alternative compared to the standard schedule to reduce the prevalence of nasopharyngeal (NP) VT pneumococcal carriage in young children with clinical pneumonia. Approximately equal numbers of infants will be enrolled in each cluster, with clusters allocated to the two groups in a 1:1 ratio.

### Study setting {9}

**Fig. 1:**
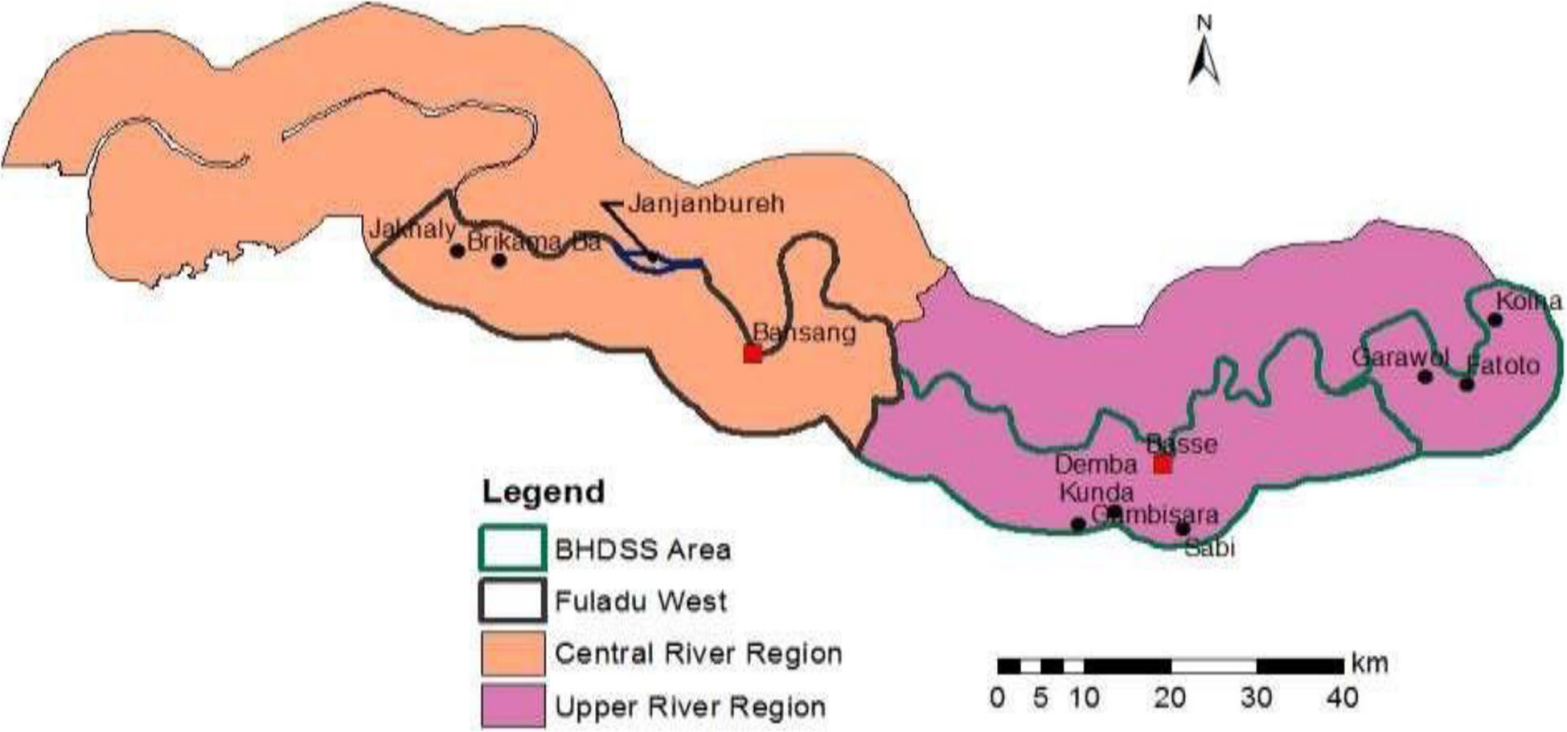
Map of The Gambia showing the trial area in the Fuladu West and Basse Health and Demographic Surveillance Areas in Central and Upper River Regions

**Fig. 2:**
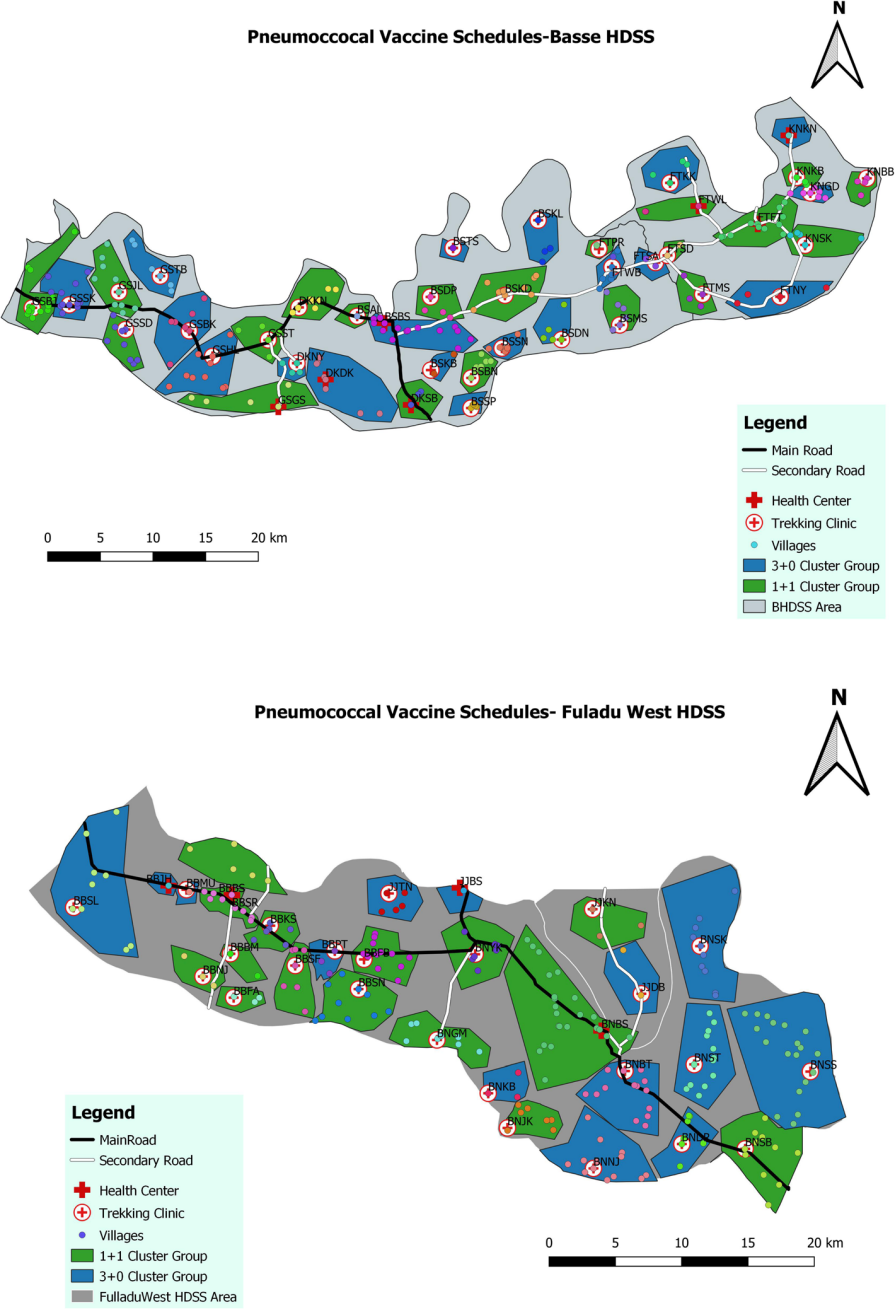
Map of the BHDSS and FWHDSS showing 68 geographic clusters of villages assigned to one vaccination clinic

PVS is being conducted in Upper and Central River Regions (URR and CRR) in the geographic area covered by the Basse and Fuladu West Health and Demographic Surveillance Systems (BHDSS and FWHDSS) (Fig. 1). The trial is based at the Basse Field Station of MRCG at LSHTM. The BHDSS population is 178,510 (225 villages) with 99,113 in the FWHDSS (213 villages); 19% of the population is aged < 5 years. The annual birth cohort in the trial area is around 10,000. The area has a child mortality rate of around 50 per 1000 live births. There are 68 geographically separate clusters of villages assigned to attend geographically separate EPI clinics (Fig. 2).

#### Participant selection

The sampling frame for selection to receive the interventions will be all infants resident in the 68 selected clusters. Residency is defined as:

- Born to or cared for by a parent or guardian who is resident for greater than 4 months as confirmed by BHDSS records or a household visit with the report of the household or compound head. Provisional residency may be established by the verbal report of the parent or guardian.
- Born to or cared for by a parent or guardian who intends to be resident for greater than 4 months with verification at a household visit and report of the household or compound head. Provisional residency may be established by the verbal report of the parent or guardian.

The sampling frame is developed and continually updated as part of the FWHDSS and BHDSS. Pregnancies and births in all households are registered from 4-monthly enumerations of each household, village reporter records, and registration at EPI clinics. These data are electronically recorded in the field and synchronised centrally on a weekly basis. A verified and updated sampling frame is available for use each week. The sampling frame lists the mother’s name, infant’s name, date of birth, father’s name, name of household head, village name, compound number and individual ID number.

### Eligibility criteria {10}

All resident infants are eligible for enrolment. Participants must meet all of the inclusion criteria and none of the exclusion criteria to be eligible.

#### Inclusion criteria

- Resident in the study area.

#### Exclusion criteria

- Intent to move out of the study area before 4 months of age
- Age greater than 9 months
- Completed PCV schedule
- Contraindication to PCV13— severe hypersensitivity to a previous dose of PCV13

For a period of 4 months at the time of initiation of the interventions, the upper age of eligibility in 3 + 0 clusters was 6 months, to enable the logistics of enrolment of approximately 1500 resident infants. In 1 + 1 clusters infants were enrolled up to 9 months of age and for a 4-month period those who had already received one, two, or three doses of PCV were eligible to receive a booster dose at 9 months of age.

### Who will take informed consent? {26a}

Firstly, we informed the community leaders in the 68 selected clusters about the nature of the trial. Trained trial staff who speak the local languages presented the trial information and answered any questions.

Eligible infants making their first presentation to the immunisation clinic after birth are identified. Trial staff determine the parent or guardian’s first language and literacy. More than three quarters of the adults in the trial area are not literate in English. Trained staff provide the trial information sheet to literate parents or guardians. If the parent or guardian is not literate, trial staff play a standardised audio recording explaining the trial in the appropriate one of four different local languages. Translation of the trial information sheet and its audio recording was validated and certified by Tostan Basse (email. gambia@tostan.org). Trained staff then enquire whether there are any questions and seek informed consent. Mothers and guardians are encouraged to discuss participation with the infant’s father before consent. Trial staff address the questions and concerns of parents or guardians. Trial staff who speak the language of the parent or guardian conduct the informed consent process. If the parent or guardian is illiterate, an impartial witness is present during the informed consent process. Each impartial witness receives the information sheet and consent form and listens to the audio consent information. Given that the impartial witnesses will see and hear standard information about the trial in their own language and that their role is to judge the voluntary nature of the participant’s decision to be enrolled, they may not be literate in English.

The consent of parents or guardians is recorded on a paper form. If literate in English the participant’s parent or guardian signs and dates the consent form. If illiterate, the impartial witness attests to the participant’s apparent understanding, that informed consent is freely given, and the responses to the specific questions on the form. Trial staff mark the participant’s responses to each of the specific questions on the form. If the parent or guardian has understood the information, they thumbprint the consent form. The impartial witness signs, or thumbprints, the consent form and dates the participant’s thumbprint. If the impartial witness is illiterate trial staff date the thumbprint of the parent or guardian and impartial witness. If a guardian provides consent, this is documented on the consent form and a statement of guardianship is obtained, with the signature of a witness if the guardian is not literate in English. The staff member obtaining consent records their name and signature on the consent form and provides an identical copy to the parent or guardian. The person obtaining consent also provides a copy of the information sheet (including the free-call contact details of two trial staff).

### Consent for collection and use of participant data and biological specimens {26b}

Consent for the collection and use of participant data and biological specimens is specified in the trial information sheet and consent form. The consent form includes specific confirmation, marked on the form and entered in the database, confirming consent for: the collection of specific numbers and types of specimens, future research using the specimens, shipping of specimens overseas, and use of unidentified data via MRCG authorised data repositories.

## Interventions

### Explanation for the choice of comparators {6b}

**Fig. 3:**
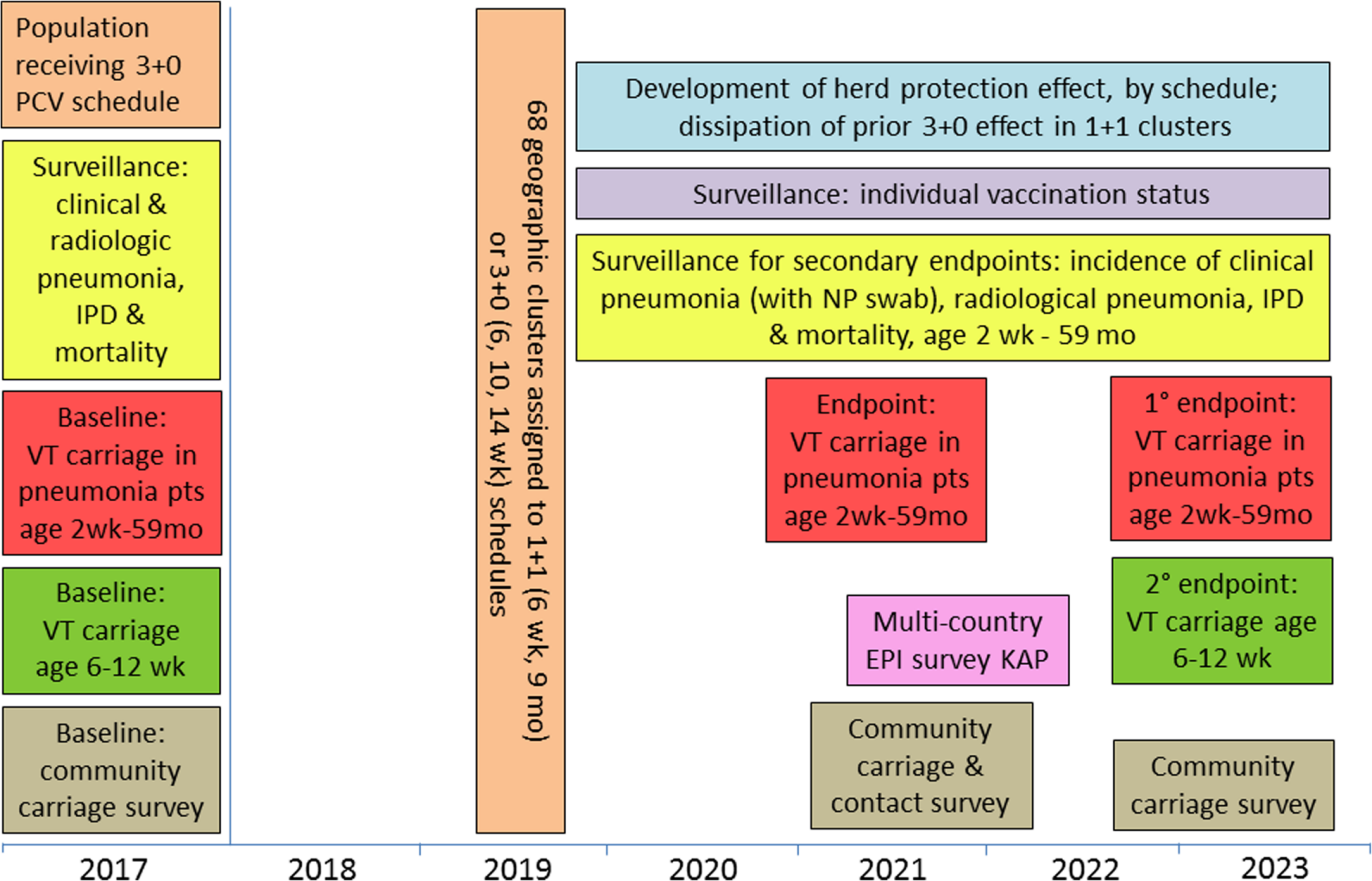
Schema showing trial groups, activities, endpoints and trial timeline

The current standard 3 + 0 schedule for PCV is well-established and has effectively controlled VT IPD and generated a degree of herd protection. Given the need to generate data that are generalizable to other African countries, PVS will implement the much more commonly used scheduling of doses at 6, 10, and 14 weeks, rather than the national schedule of 2, 3, and 4 months. The alternative 1 + 1 schedule (doses scheduled at ages 6 weeks and 9 months) will schedule its first dose at the same age as the 3 + 0 schedule. The booster dose at 9 months of age is a standard EPI visit in low-income countries, and in Africa measles and yellow fever vaccination are scheduled at this visit. The age of 9 months is the earliest at which the booster dose could be incorporated into already established EPI schedules in low-income countries and takes advantage of a key EPI visit to optimise coverage of administration in the population. We hypothesise that the 1 + 1 schedule will provide adequate direct protection between 2 and 9 months of age, during which time the risk of VT disease in our setting is very low, and potentially superior herd protection and similar overall effectiveness compared to the 3 + 0 schedule. The interventions will be delivered for 4 years (Fig. 3). We will measure the primary and secondary endpoints in Year 4 of intervention delivery, at which time we assume the differential effects of the two schedules will have stabilised [32–34].

### Intervention description {11a}

The intervention was initiated at the same time in all clusters across the trial area. The experimental intervention in this trial is the scheduling of PCV13 for infants resident in geographic clusters of villages with the first dose due at 6 weeks of age and a booster dose scheduled at 9 months of age. The standard intervention is the scheduling of PCV13 for infants with doses due at 6, 10, and 14 weeks of age. There are few data to indicate the period of time required for the dissipation of the population-level effects of a PCV schedule while transitioning to a new schedule. Data from PSP in rural Gambia suggest that, in the 2-year period following the introduction of PCV13, herd effects were developing as the incidence of PCV13 only serotype IPD in the 2–4 years age group had fallen from 50 per 10^5^ person-years in 2010/2011 to 20 in 2013, despite coverage of PCV13 in mid-2013 being only 10% in this age group [9]. At the time the intervention was initiated, the 1 + 1 schedule was implemented with catch-up of the booster dose for infants up to 9 months of age who had already received two or three doses of PCV, the rationale being that this would be the approach if countries were to transition from a 3 + 0 schedule to a 1 + 1 schedule, and not waste doses of PCV. Catch-up of the booster dose was implemented for 4 months and then ceased.

PCV13 vaccine is licenced in many countries and has been approved for use in The Gambia since 2011. The EPI procures the vaccine through global systems coordinated by UNICEF. This trial delivers PCV13 in collaboration with, and through the structures of the Gambian EPI, and under the operational conditions of the public health system. The EPI receives PCV13 into a central cold-storage facility. Vaccine is transported to the regional centres either by a specially designed EPI ‘cold van’ or by cold storage units carried by Regional Health Directorate (RHD) vehicles. The RHDs in URR and CRR are based in Basse and Bansang respectively. The URR RHD stores vaccine in solar refrigerators in Basse and the CRR RHD stores vaccine in a purpose-built ‘cold room’ in Bansang. From Basse and Bansang, small volumes of vaccine are distributed on a monthly basis to eight ‘base clinics’ in Basse, Gambisara, Demba Kunda, Fatoto, Koina, Sabi, Bansang, and Brikamaba. These base clinics carry small volumes of vaccine to each of the EPI clinics in the 68 different geographic locations involved in PVS. Solar vaccine refrigerators are used for storage at the base clinics. The trial uses the existing EPI procedures to monitor and record the vaccine accountability and cold chain documentation with daily logs.

Immunisation is undertaken at the 11 fixed health centres in the study area on one to two days each week. Mobile clinics in the 57 other sites are held once or twice per month. Given the frequency of EPI clinics, there is generally some delay in the time that vaccines are actually received. Administration of PCV13, and other antigens, by EPI staff will follow the schedule allocated for the particular geographic cluster (Table 1), apart from when a list of exceptions dictate that an infant receives the schedule of the other group and not the locally allocated schedule (Table 2). In 2016 coverage in the BHDSS of three doses of PCV at 12 months of age was 92% and coverage of one dose of measles vaccine was 82% with measles coverage of 92% by 18 months of age.

**Table 1:** Vaccination schedule in the Pneumococcal Vaccine Schedules (PVS) trial

Age	1 + 1 schedule			3 + 0 schedule	
	PCV13	Other antigens		PCV13	Other antigens
At birth or soon after	No	BCG, OPV 0, Hep B		No	BCG, OPV 0, Hep B
6 weeks	PCV13	Penta 1, OPV 1, Rota 1		PCV13	Penta 1, OPV 1, Rota 1
10 weeks	No	Penta 2, OPV 2, Rota 2		PCV13	Penta 2, OPV 2, Rota 2
14 weeks	No	Penta 3, OPV 3		PCV13	Penta 3, OPV 3
Nine months	*PCV13	MR, YF, OPV 4		No	MR, YF, OPV 4
Twelve months	**	MenA, DTP booster		No	MenA, DTP booster
Eighteen months	**	MR, OPV5		No	MR, OPV 5

*For a 4-month period after the initiation of the intervention, infants allocated to 1 + 1 who had already received two or three doses received a dose of PCV13 at age 9 months

**PCV13 booster may be administered beyond the scheduled age if not given at age 9 months

**Table 2:** Circumstances when infants will not receive the locally allocated schedule for PCV13

Locally allocated schedule	Reason not to receive locally allocated schedule
3 + 0	Infant resident in 1 + 1 cluster and visiting a 3 + 0 clinic Infant completed 1 + 1 schedule and migrated to 3 + 0 village
1 + 1	Infant resident in 3 + 0 cluster and visiting 1 + 1 clinic Infant completed 3 + 0 schedule and migrated to 1 + 1 village Declined or withdrawn consent Non-resident

### Criteria for discontinuing or modifying allocated interventions {11b}

Trial participants are discontinued from participation in the study if:

- Any clinically significant adverse event, intercurrent illness, or other medical condition or situation occurs such that continued participation in the study would not be in the best interest of the participant.
- The parent or guardian so desires.

Participants who attend an EPI clinic outside their cluster and within the study area, but continue to reside in their original cluster, receive the trial schedule originally indicated on their infant welfare card and on the trial sticker. If participants migrate within the study area before completing their PCV schedule they continue to receive the trial schedule allocated in the cluster of their new residence (Table 2). Participants who migrate after completing their PCV schedule do not receive any further doses of PCV in the cluster of their new residence (Table 2). Parents in the 1 + 1 group are advised that if they migrate permanently out of the trial area they should attend the next available EPI clinic to complete the standard schedule for PCV. If an infant allocated to the 1 + 1 group visits an EPI clinic outside the study area the parent is instructed to request that their child receive the trial schedule indicated on the infant welfare card and study sticker. The study sticker includes free-call telephone numbers so that parents, or EPI staff outside the study area, may call for guidance. Infants resident in the alternative schedule clusters who decline consent are assigned to the national standard schedule.

### Strategies to improve adherence to interventions {11c}

Adherence to the trial vaccination schedules is facilitated by exclusion criteria including an intention to migrate out of the study area before 4 months of age. Migration out of the trial area is limited; on average, only 10 children per cluster per year migrate out of the area. Also, lists of infants allocated to the 3 + 0 group who have not completed three doses by the age of 5 months, and lists of infants allocated to the 1 + 1 group who have not received the booster dose by the age of 11 months, are generated every month to guide defaulter tracing at home visits throughout the study area.

### Relevant concomitant care permitted or prohibited during the trial {11d}

PCV13 has been co-administered with Measles-Mumps-Rubella [35] and Measles-Mumps-Rubella-Varicella [36] vaccines but the results of both these studies do not report investigations of potential interference between the vaccines. PCV10 (Synflorix®) has been co-administered with yellow fever vaccine in a study of an investigational GSK vaccine although investigations of potential interference between the vaccines have not been published. A different investigational PCV10 vaccine manufactured by the Serum Institute of India has been co-administered with yellow fever vaccine with non-inferior immune responses [37]. Studies of yellow fever vaccine co-administration with polysaccharide protein-conjugate quadrivalent meningococcal vaccine had not detected any adverse interaction [38]. The investigators are not aware of any data, or ongoing studies, that evaluate potential immune interference with the co-administration PCV13 and yellow fever vaccine. An accompanying paper describes the protocol for a related trial in which we are investigating the effect of co-administration of PCV13 and yellow fever vaccine.

### Provisions for post-trial care {30}

The trial may be stopped early if there is evidence that the risk of pneumococcal disease is greater in one compared to the other trial group. If the Data Monitoring Committee (DMC) recommends that the trial be stopped early, a joint meeting of the DMC, Trial Steering Committee (TSC) and Central Stakeholder Committee will make a recommendation to the Sponsor regarding post-trial procedures, including whether a dose of PCV be administered to children in a group found to have inferior PCV-induced protection. LSHTM carries clinical trial/non-negligent harm insurance and medical malpractice insurance applicable to this trial.

### Outcomes {12}

The outcomes in the trial and the timing of their measurement are shown in Fig. 3. The primary trial endpoint is:

- NP carriage of VT pneumococci in children aged 2 weeks–59 months with clinical pneumonia presenting to health facilities in the study area (measured in year 4).

Secondary endpoints will be:

- NP carriage of non-vaccine type (NVT) pneumococci in children aged 2 weeks–59 months with clinical pneumonia presenting to health facilities in the study area (measured in year 4).
- Population-based and age-stratified NP carriage prevalence of VT and NVT pneumococci (measured in years 2 and 4).
- NP carriage of VT and NVT pneumococci in infants presenting for the first dose of PCV aged 6–12 weeks (measured in year 4).
- Incidence of radiological pneumonia in children aged 2 weeks–59 months (safety endpoint).
- Incidence of clinical pneumonia in children aged 2 weeks–59 months (safety endpoint).
- Incidence of clinical pneumonia associated with NP carriage of VT and NVT pneumococci in children aged 2 weeks–59 months (safety endpoint).
- Incidence of serotype-specific IPD in children aged 2 weeks–59 months (safety endpoint).
- Incidence of hospitalisation in children aged 2 weeks–59 months (safety endpoint).
- Mortality rate in children aged 2 weeks–59 months (safety endpoint).

#### Justification of outcomes

Measuring the prevalence of VT pneumococcal carriage in children with pneumonia and in the community are effective methods to determine the population-level effect of PCV schedules on pneumococcal transmission. The primary endpoint relates to the important public health condition of clinical pneumonia in children and the associated VT pneumococcal carriage correlates with community-level transmission. This primary endpoint may be replicated in other settings that investigate the transition from a standard to alternative PCV schedule. The primary endpoint will be measured in Year 4 when the differential effects of the two schedules have stabilised [32–34]. To explore potential early impact of the alternative schedule, we will conduct a secondary exploratory measurement of VT carriage prevalence in children with clinical pneumonia in year 2, specifying a superiority hypothesis (Fig. 3).

Surveys of pneumococcal carriage in the community have little selection bias as all residents of all ages are included in the sampling frame. Compared to VT prevalence in children with clinical pneumonia, the community-level VT carriage measurement is less prone to information bias as participants do not need to meet the clinical pneumonia criteria to have a NP swab. Cross-sectional surveys are a quick and relatively inexpensive way to measure VT carriage in the study population and give a broad scope of the effects of the two PCV schedules in different age groups. The population survey of VT carriage in year 2 will allow an exploratory measurement of any immediate impact from the alternative PCV schedule. The year 2 survey will be completed in parallel with a survey of interpersonal contact patterns. The survey in year 4 will provide an important secondary endpoint evaluating the effect of the alternative schedule in the population as children receiving the alternative schedule age and accumulate within the population.

In order to isolate the herd effects of the schedules in very young infants, the secondary endpoint of VT carriage prevalence in infants aged 6–12 weeks presenting for their first dose of PCV13 will be measured in a cross-sectional manner in year 4 (Fig. 3). Safety endpoints covering the incidence of clinical pneumonia, radiological pneumonia, IPD, hospitalisation and mortality will be measured throughout the 4 years of the trial (Fig. 3). The incidence of clinical pneumonia associated with NP carriage of VT and NVT pneumococci will also be measured.

The dynamics of age-dependent VT pneumococcal transmission are complex and vary by population, contact patterns and the related carriage data have important implications on accurate vaccine protection estimates [39, 40]. Including an interpersonal contact pattern survey in Year 2 parallel with the survey of VT carriage will generate data on contact patterns for planned mathematical modelling of the effect of alternative PCV schedules. The parallel surveys will give insight into the magnitude of association between age-related carriage prevalence and rates of interpersonal contact and whether infection is associated with physical or non-physical contacts.

The emergence of NVT serotypes causing IPD in both children and adults following the introduction of PCV13 has been a source of concern. The effect of an alternative schedule on serotype replacement dynamics has not been well described. The measurement of NVT pneumococcal carriage in the population in years 2 and 4 will provide data on the effect of the alternative schedule on NVT pneumococcal transmission

### Participant timeline {13}

**Fig. 4:**
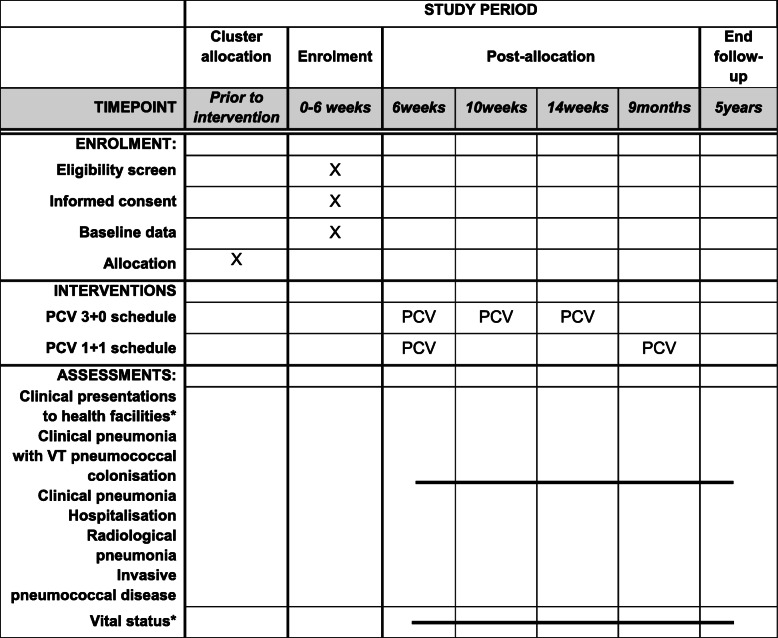
Adapted SPIRIT figure showing the timeline of cluster allocation, enrolment, intervention, and assessments for infants enrolled at EPI clinics. PCV13 (PCV) – 13 valent pneumococcal conjugate vaccine; 3 + 0 – standard schedule of three primary doses; 1 + 1 – alternative schedule of one primary dose and one booster dose. *VT* vaccine-type pneumococcal serotype. *Clinical presentations to health facilities and vital status are under continuous surveillance among infants enrolled at EPI clinics to receive the interventions and among all resident children < 5 years of age

Participants in the trial are enrolled in one of the two groups (Table 1, Fig. 3) with the timing of specimen enrolment, interventions, and assessments shown in Fig. 4.

### Non-inferiority margin

We determined the non-inferiority margin using the concept of ‘the largest loss of impact of the current treatment that would be clinically acceptable’, an approach recommended by the US FDA [41]. The non-inferiority margin uses a relative metric as the relative effect of the standard schedule is likely to be constant. The introduction of the 3 + 0 schedule has reduced NP VT prevalence in children from 47 to 13%, i.e. a 72% reduction (author’s own data). Baseline risk may change over time, so an absolute value for the non-inferiority margin may not provide a reliable measure of the difference in impact of the two schedules.

Given the important advantages of the 1 + 1 schedule, the non-inferiority margin will be a 15% loss of the impact of the 3 + 0 schedule; this value is supported by empiric survey data. An online survey was sent to 72 individuals involved in pneumococcal vaccine research, policy and clinical care. Valid responses were received from 19 respondents. The survey question presented 10 hypothetical results of VT prevalence in the two trial groups, on a scale of increasing ‘loss of impact’ associated with the 1 + 1 schedule, from a baseline of no loss (i.e. a 0% loss of impact) in the 1 + 1 versus 3 + 0 group, up to a 50% loss of impact. Respondents were asked to consider themselves as decision-makers in their national immunisation programmes requested to consider a change to their national programme from a 3 + 0 to 1+ 1 schedule based on the results of the trial. Respondents selected one option corresponding to the ‘loss of impact’ which would sway their decision against introducing the new 1+ 1 schedule. Using the metric of a percentage loss of impact of the 3 + 0 schedule which would sway decisions against the new schedule the mean value was a 22% loss of impact. The results of the survey support our decision to use a non-inferiority margin that the 1 + 1 schedule be associated with a ≤15% loss of the total impact of the 3 + 0 schedule.

The prevalence of NP carriage of VT pneumococci from Jan–Sept 2015 was 17% and fell to 15% in Oct–Dec 2015 (author’s own data). Assuming VT prevalence of 13% in the 3 + 0 group at the beginning of the trial, a 15% loss of impact translates to prevalence in the 1+ 1 group of (1 − [0.72 − (0.72 × 0.15)]) × 47% = 18.3%. We used changes in VT prevalence to inform the non-inferiority margin because reductions in VT carriage following the introduction of PCV can predict the impact of PCV on IPD and because carriage prevalence is proportional to the rate of pneumococcal transmission in the population [42].

For the secondary endpoints of community pneumococcal carriage, the non-inferiority margin of a ≤15% loss of impact in the 1+ 1 compared to the 3+ 0 schedule clusters, will be used as described above. The same VT prevalence will be assumed within the community.

### Sample size {14}

Sample size calculations were based on the primary outcome of NP carriage of VT pneumococci in children aged 2 weeks–59 months with clinical pneumonia. The primary outcome will be measured in random samples of participants in each of the 68 clusters (if a minimum of 60 participants were sampled in each cluster there would be 1980 in each arm and 3690 in total). Using Basse data from 2015, we assumed that the prevalence of VT carriage to be 13% in both the 3 + 0 and 1 + 1 arms. Based on the non-inferiority assumptions presented in the previous section, the 1 + 1 schedule will be considered non-inferior if the upper limit of the one-sided 95% confidence interval for the prevalence ratio is ≤1.38 (0.18/0.13 = 1.38). Using Basse data, we estimated the intra-class correlation (ICC) to be 0.01–0.02. We calculated study power using the methods of Farrington et al. [43] and Donner et al. [44]. Using the more conservative value for ICC of 0.02, a minimum number measured for the endpoint per cluster of 60, 33, and 35 clusters per arm (with design effect of 2.18) and alpha=0.05, a sample size of 1980 per arm will provide 93% power to test the non-inferiority of the 1 + 1 schedule.

To increase confidence in the power of the study we simulated trial data using 2015 data from the BHDSS based on cluster-wise VT carriage in children with pneumonia. The simulated data were designed to be similar to the villages included in this trial with respect to the mean carriage prevalence, as well as the variability in prevalence across clusters. Using 1000 simulated populations, each consisting of 68 clusters of 60 individuals, baseline prevalence of 10% and a largest acceptable increase of 4%, i.e. from 10 to 14%, study power was ≥85% in most scenarios.

As opposed to the non-inferiority hypothesis that is specified in year 4 of the study we will specify a superiority hypothesis for the measurement in year 2 of VT prevalence in children with clinical pneumonia. Setting a 5% level of significance and power of 80%, with ICC=0.02 and assuming the prevalence of VT carriage in the control group is 18%, we wish to detect an absolute difference of 8%. That is, the smallest clinically significant difference that we wish to detect is equivalent to 18% versus 26% prevalence in the two groups. Given these parameters, if we measure the endpoint in 20 individuals per cluster, we would need to include 29 clusters in each group, that is, a total of 58 clusters. We will measure the endpoint in 68 clusters.

Study power for the secondary endpoint of VT prevalence in infants aged 6–12 weeks presenting for their first dose of PCV is based on the same assumptions as for the primary endpoint and study power will be similar to that for the primary endpoint.

In the community carriage surveys in years 2 and 4, we will sample 60 residents in each of the 68 clusters. The same assumptions and methods of power calculation as for the primary endpoint were used. We assume ICC=0.02, 15% baseline VT carriage prevalence in both clusters, and a ≤1.38 prevalence ratio derived from the ≤15% NI margin. If the upper bound of the 95% confidence interval of our prevalence ratio comparing the 1 + 1 to the 3+ 0 clusters is ≤1.38, then the alternative schedule will be considered non-inferior. Following these assumptions, the community carriage surveys in years 2 and 4 will have power > 94% if we collect 4080 samples evenly across 68 clusters (i.e. 60 per cluster).

For the interpersonal-contact pattern survey in year 2, 1500 participants from the carriage study will be enrolled. Recruiting 1500 participants will enable us to detect an absolute difference of 1 mean contact per day between age groups, with a 5% significance level, and 90% power. These calculations are based on the work of le Polain de Waroux et al. [45]. The study is not powered to determine non-inferiority for other secondary or safety endpoints.

### Recruitment {15}

All infants resident in the BHDSS or FWHDSS are eligible for enrolment in the PVS field trial. When infants present to immunisation clinics, trial staff confirm their identity and apply the trial inclusion and exclusion criteria. The mother/guardian of infants who meet the inclusion criteria and do not have exclusion criteria are informed about the nature of the trial as part of the informed consent process. The screening of infants and recruitment of participants is electronically entered in real-time into the trial data management system. A trial sticker is fixed to the child welfare card and the intervention schedule is marked on the child welfare card. Enrolment to receive the 1 + 1 intervention will cease 3 months prior to the close of clinical surveillance at the end of year 4 of the study.

Children aged 2 weeks to 59 months who are resident in the study area are under surveillance for clinical endpoints. Children who present unwell to health facilities in the study area are evaluated for criteria indicating the need for clinical investigation.

The cluster-wise selection of infants aged 6–12 weeks at the first dose of PCV13 for laboratory analysis of NP swabs will include all individuals in clusters with a number of individuals below a threshold number set for statistical efficiency. In clusters with numbers greater than the threshold, selection will follow a random scheme within each cluster. Patients will be randomly sampled within clusters from those born in the trial area, across age, time and space.

For the community surveys of pneumococcal carriage, residents of all ages will be included in the sampling frame. The HDSS will be used to determine cluster, village, and household population sizes and as a reference for the members of a household. Three-level cluster sampling using probability proportional to size will be used to select 2 villages within each of 68 clusters, with three households per village and 10 individuals per household. Ten individuals will be randomly selected from each household following the age-structure ratio 2:2:2:2:1:1 (0–11 months, 12–23 months, 2–5 years, 6–14 years, 15–44 years, 45 years and greater). Individuals not listed as resident within a selected household may be eligible if confirmed by the head of household as resident in the past 4 months.

## Assignment of interventions: allocation

### Sequence generation {16a}

Sixty-eight PVS trial clusters were randomised using a blocked scheme to ensure similar numbers of clusters were assigned to each group. Randomisation was stratified by a binary variable correlated with ‘high’ or ‘low’ cluster-level incidence of clinical pneumonia. Randomisation was carried out in permutations using the above stratification until selections achieved balance in terms of presence of a health centre and balance on population size between the two groups. In order to select the stratifying variable of cluster-level incidence of clinical pneumonia, cluster-level prevalence of ‘high’ or ‘low’ VT carriage in children with clinical pneumonia in the BHDSS was correlated with: clusterwise population density, rates of hospitalisation, clinical pneumonia, radiological pneumonia, IPD and mortality. Of the five listed outcomes, clinical pneumonia incidence had the closest correlation with VT carriage prevalence and thus was chosen as the stratifying variable.

### Concealment mechanism {16b}

A public event was held to announce group allocations of each cluster of villages in the PVS trial area. Representatives of each cluster were present at the public event. Selection of the randomisation list at the public event involved random selection of one of 100 valid randomisation lists. Thus, the investigators and cluster representatives had no knowledge of the allocation sequence at the time of group allocation.

### Implementation {16c}

An independent statistician prepared the cluster randomisation lists while trial staff and cluster representative were informed of the allocation as described in the previous section.

## Assignment of interventions: blinding

### Who will be blinded {17a}

Vaccinators and parents may be aware of the schedules used. Laboratory staff are blinded as specimens are labelled with a unique identification number that does not identify the study group. Blinding of laboratory staff will avoid bias given the laboratory-based measurement of the primary endpoint. Statisticians will analyse data in a pseudo-blinded fashion with the two groups identified by an indicator label rather than the identity of each group.

### Procedure for unblinding {17b}

Given that participants are not blinded to their group allocation, a procedure for unblinding is not needed.

## Data collection and management

### Plans for assessment and collection of outcomes {18a}

Resident children aged 0–59 months are under passive surveillance for clinical events. For all children presenting to a health facility in trial area staff provide standardised evaluation, investigation and recording of episodes in an electronic medical record. Data collection forms are not included in the protocol but are available on request.

Each child presenting unwell to a health facility in the trial area is registered, and if resident and aged 0-59 months, is eligible to be screened for clinical pneumonia. Whenever possible, a different staff registers attendance and conceals the infant welfare card from the staff performing clinical pneumonia screening (to minimise potential bias due to knowledge of the residential location and group allocation). Trial nursing staff screen for clinical pneumonia by determining if there is a history of cough or difficulty breathing, the respiratory rate, peripheral arterial oxygen saturation, and presence of lower-chest wall indrawing (Table 3).

**Table 3:**
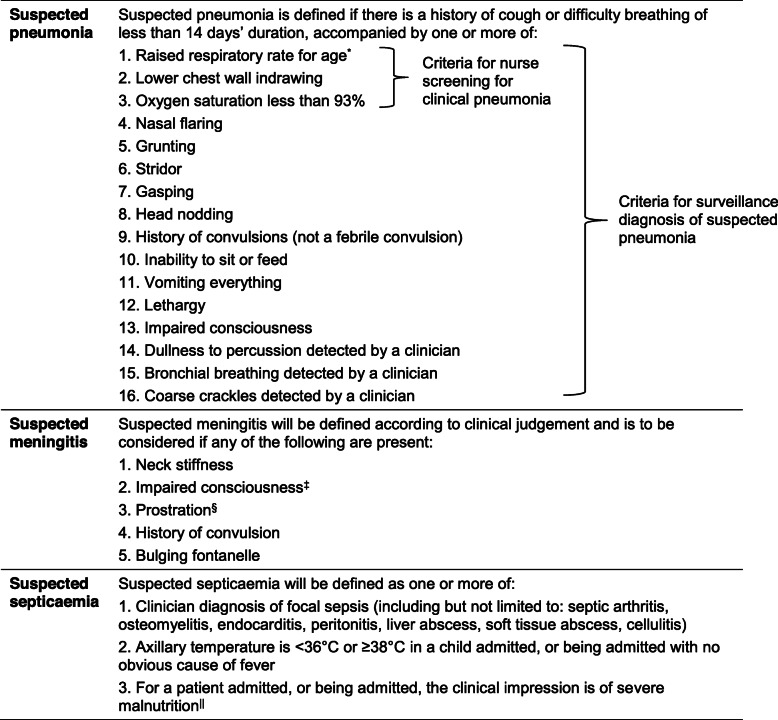
Clinical definitions for suspected pneumonia, septicaemia, and meningitis

Cough or difficulty breathing accompanied by one or more of the signs numbered 1, 2, and 3 is used to define clinical pneumonia at nurse screening

Cough or difficulty breathing accompanied by one or more of the signs numbered 1–8 is used to define suspected pneumonia as the surveillance diagnosis by nurses in outlying clinics and doctors in Basse and Bansang

*Raised respiratory rate for age is defined as greater than or equal to 60 breaths per minute for children less than 60 days of age, greater than or equal to 50 breaths per minute for children aged at least 2 months but less than 12 months, and as greater than or equal to 40 breaths per minute for children at least 12 months but less than 60 months. If the respiratory rate is raised on the first measurement it will be measured again after at least 5 min- and deemed raised if still greater than the given age-specific threshold

^‡^Impaired consciousness is defined as V, P, or U on the AVPU score, where A is if the patient is alert, V if responsive to verbal stimulus, P if responsive to pain stimulus, and U if unresponsive

^§^Prostration is defined as inability to feed or to remain in a seated position in a child otherwise able to do so

^||^Severe malnutrition is defined according to the WHO definition

Trial nursing staff use standardised criteria (Table 3) to classify patients according to a diagnosis of suspected pneumonia, septicaemia, and/or meningitis or other diagnoses consistent with the scheme of the Integrated Management Childhood Illness (IMCI) guideline. Patients are referred to clinicians in Basse and Bansang if there is suspected meningitis, hypoxia, or other severe illness requiring a higher level of care. According to standardised diagnosis patients are investigated as per the guideline in Table 4. Radiographs are performed in Basse, Bansang.

**Table 4:** Guideline for investigation of patients

a. Patients admitted with any acute medical problem will have a blood culture taken, a plasma aliquot stored, a rapid malaria test done and haemoglobin measured. Samples will not be collected from children admitted electively or those with surgical problems, trauma, acute burns or non-infectious neonatal problems. b. Patients with suspected meningitis will have a lumbar puncture and chest X-ray done. c. Patients with clinical pneumonia will have a NP swab. If admitted to hospital with clinical or suspected pneumonia, patients will have a chest X-ray, NP swab and blood culture. d. Patients with suspected septicaemia without a focus will have a blood culture and chest X-ray done. e. Patients with suspected septicaemia with a focus will have a blood culture only. f. Lung aspirate will be considered if large peripheral consolidation is demonstrated by X-ray. g. Other investigations, including pleural aspiration, may be considered according to clinical indication.	

Fatoto and Jakhaly. Patients in Koina and Garawol are transported to Fatoto for X-ray and then returned for treatment. Patients in Gambisara and Demba Kunda are transported to Basse for X-ray. Patients in Janjanbureh are transported to Bansang for X-ray. Patients in Jakhaly are transported to Brikamaba for X-ray.

For resident patients with clinical pneumonia treated as an outpatient, or clinical or suspected pneumonia treated as an inpatient, a NP swab is taken (Table 4). We conduct frequent supervision of case ascertainment procedures and quality control of patient evaluation and NP swab technique. All children admitted are investigated according to the standardised guide in Table 4. Any child admitted with an acute medical problem has a blood culture taken, a plasma aliquot stored, a rapid malaria test done and haemoglobin measured. Samples are not collected from children admitted electively or those with surgical problems, trauma, acute burns or non- infectious neonatal problems. Participants admitted with suspected sepsis defined according to standardised criteria will have a blood culture done and those with suspected meningitis a blood culture and lumbar puncture. For those admitted with clinical pneumonia, or a danger sign, or focal chest signs, a blood culture and chest X-ray will be performed and pleural fluid or lung aspirate obtained as clinically indicated. Other investigations are done according to the clinical judgement of the attending clinician.

Radiological pneumonia is defined as radiological end-point consolidation as per the procedure for classification according to the WHO standard [49]. If multiple radiographs are taken during an episode of illness the worst radiographic appearance in the 3 days following the date of screening will be accepted as final. Pneumococcal pneumonia will be defined as a patient with clinical pneumonia accompanied by isolation of *S. pneumoniae* from a normally sterile site. Pneumococcal radiological pneumonia will be defined as WHO-defined end-point consolidation accompanied by isolation of *S. pneumoniae* from a normally sterile site. We will classify pneumococcal pneumonia as being caused by a) PCV13 vaccine serotypes or b) non-PCV13 vaccine serotypes. Non-typeable isolates will be included as a separate group. Episodes will be considered as separate events if the first and subsequent consultations are at least 30 days apart, or if a pneumococcus of a different serotype is isolated during each episode. Cases of pneumococcal pneumonia in which two different serotypes are isolated will be classified as one case of pneumococcal pneumonia, but two different episodes of serotype-specific IPD if the two different serotypes belonged to different serotype categories. Hypoxic pneumonia will be defined as clinical pneumonia with peripheral O_2_ saturation < 93%.

### Plans to promote participant retention and complete follow-up {18b}

The informed consent process allows time for consideration by both parents and discussion among family members. We provide participants with a trial information sheet that includes free-call phone numbers that can be used at any time. We provide instructions to participants if attending an EPI clinic outside the trial area that facilitates administration of vaccine according to the trial schedule.

At each visit, trial staff identify the infant and the group allocation, based on the trial sticker on the infant health card, residential location, and electronic vaccination record. Trial staff routinely record whether infants are migrating, or intend to migrate, within or out of the trial area. Administration of PCV by EPI staff follows the schedule allocated on the infant health card. Following administration of a dose of PCV, the date is recorded on the health card and in the electronic vaccination record.

Attrition bias due to the dropout of clusters is unlikely as this will require all five or six villages in a cluster to withdraw. Migration out of the trial area is limited; on average, only 10 children per cluster per year migrate out of the area. If clusters drop out, study power may be retained by increasing the number measured for the endpoint in each cluster. We will avoid the withdrawal of villages through close engagement with village representatives and continual informed consent.

### Data management {19}

A data management plan has been prepared and approved by the MRCG Head of Data Management and is available on request. Data are collected on eCRFs using a standardised format. Electronic data capture is done offline using encrypted devices which is then synchronised with a central server on a weekly basis. Trial staff attend all EPI clinics, confirming the identity of all infants and recording immunisation data in real-time. Trial staff generate source data on electronic devices at regular visits as per the trial timeline.

Data entered into eCRFs is monitored for completeness and consistency against the relevant source documents. Independent trial monitors undertake 100% verification of source data for the primary endpoints and informed consent. Anomalies identified are reconciled with the source.

Front-end data quality checks are programmed into the data capture application. Backend edit checks and validation checks are built-in to monitor the validity of data (e.g. to identify inconsistent dates and times, and clinical and antropometric measurements outside defined ranges, etc.). Data queries are generated weekly by the data manager for resolution by trial staff. Reports of data quality are generated periodically.

The trial database is housed on a secure network server with restricted access to the backend. The backend comprises a MSSQL database which will be regularly backed up as part of the organisation’s disaster recovery plan. An in-house web-based application is used for the database structure, using the PHP\ASP.Net platform connecting to the MSSQL backend database. A recovery point objective is set so that systems and data will be restored back 24 h prior to a failure. Daily and monthly backups of all media servers are done to achieve this objective.

The e-CRF is used as the specification for the design of the database. An annotated CRF indicates the relationship between the variable names in the database and the fields in the CRF. A data dictionary uses a standard template, including a list of database variable names, variable descriptions, data types, and sources, valid values, and in-built edit checks. Standard data coding will be used (e.g. latest MedDRA version accessed through the internet will be used for adverse event reporting).

The long term storage of research records is done in accordance with MRCG policies and procedures for archiving. All paper records will be held for at least 10 years in the Unit’s archiving facility. Data in electronic format will be held indefinitely on our Electronic Data Repository. The trial is run in compliance with the MRC Corporate Information Security Policy [50] and the Unit’s Information and Communication Technology Security Policy. The study is conducted in compliance with the ICH Harmonized Tripartite Guideline for Good Clinical Practice E6 (R2 Step 4).

### Confidentiality {27}

No subject identifiable information (names, addresses etc.) is entered into the trial database. Identifiable information is stored in the electronic medical record, vaccination and BHDSS databases, encrypted and password-protected, and accessible to limited staff involved in the care of patients. Trial monitors only access pre-specified, non-identifiable data. Informed consent documents are stored in locked, fire-resistant filing cabinets to which only the Principal Investigator and a limited number of delegated clinical trials personnel have access. Data are backed up at the end of each day in the field on encrypted flash drives to prevent any data loss during transit. All computers within MRCG are access controlled with strong password policies that prevent unauthorised access to networked user machines. Users to whom network access has been given are granted necessary privileges to the trial database based on their trial roles.

### Plans for collection, laboratory evaluation and storage of biological specimens for genetic or molecular analysis in this trial/future use {33}

The trial collects blood and NP specimens but procedures do not include genetic or molecular analyses of human material.

NP specimens are collected using flocked nylon swabs inserted into storage media which are then placed in a dry shipper for later transport to Basse. On isolated occasions when shippers are unavailable in the health facility, NP specimens are transported to the MRCG laboratory in Basse within 6 h. To culture NP specimens, a 10 μl loop of vortexed NP specimen is inoculated onto blood agar with 5% gentamicin and incubated in CO_2_. Identification of pneumococcal colonies follows recommended methods [46]. Specimens positive for pneumococcus undergo latex sweep serotyping in Basse. Morphologically different pneumococcal colonies are selected from the primary plate, purified, and stored. For internal quality control (QC), a proportion of NP specimens is processed by two different operators and results compared. External QC on sweep serotyping involves blind assessment of known spiked samples, prepared by the Murdoch Children’s Research Institute (MCRI) Pneumococcal Laboratory, placed among the routine specimens delivered to the laboratory. As further validation of the latex sweep serotyping, a proportion of the specimens that are positive for pneumococcus will be subjected to molecular serotyping microarray analysis by BUGS Bioscience at St George’s, University of London (SGUL). Microarray is the most sensitive method for detecting carriage of multiple pneumococcal serotypes; its specificity is similar to high-quality latex sweep [47]. The Basse laboratory undergoes external quality control for bacteriology according to One World Accuracy International (Burnaby, British Columbia, Canada).

An event of VT colonisation is defined as detection of pneumococcus in a NP specimen including serotypes 1, 3, 4, 5, 6A, 6B, 7F, 9 V, 14, 18C, 19A, 19F, or 23F, using latex sweep methods. All other serotypes will be defined as non-VT. Non-typeable isolates are defined as pneumococcal by colony morphology and biochemical means, or inability to serotype by the Quellung method.

According to the guideline for investigation, blood is collected for a rapid malaria test (0.02 ml), rapid haemoglobin measurement (0.02 ml), whole blood for plasma aliquot (1–2 ml), and blood culture (at most 3 ml). Obtaining 3 ml of blood in the blood culture optimises the detection of bacteraemia and thus the monitoring of safety in the trial. Blood will be collected using sterile technique and inoculated into culture bottles (Bactec Peds Plus). An automated system (Bactec 9050, Becton Dickinson) will be used for blood cultures. Bottles that signal positive will be sub-cultured onto blood agar, chocolate agar, and McConkey agar. Bottles which fail to signal within 5 days will be considered negative. Isolates grown will be identified using conventional microbiological techniques and biochemical tests (API, Biomerieux). Other sterile site samples will be processed using standardised techniques [48]. *S. pneumoniae* will be identified by colony morphology, susceptibility to ethylhydrocupreine and, if susceptibility is equivocal, by bile solubility, and reaction with polyvalent antisera (Statens Serum Institut, Copenhagen, Denmark). Isolates classified as contaminants will include coagulase-negative staphylococcus, bacillus species, micrococcus species, and *Streptococcus viridans*. Plasma aliquots will be stored and tested for antibiotic activity using a fully sensitive bacterial control strain. Invasive pneumococcal isolates are serotyped using a latex agglutination assay which employs factor and group-specific antisera (Statens Serum Institut, Copenhagen, Denmark).

## Statistical methods

### Statistical methods for primary and secondary outcomes {20a}

Coverage of each schedule during the course of the trial will be reported at the end of each of the four annual periods, as the proportion of surviving, resident children, who have completed the respective schedules at the ages of 12 and 18 months. Coverage calculations will be classified as receiving the respective schedules in a timely (completed before age 7 months [3 + 0] or 13 months [1 + 1]) or delayed fashion. Cross-over between groups will be defined as (a) change of residential location from a 1 + 1 to 3 + 0 cluster, and vice versa, for a period of 4 or more months and (b) receipt of the third dose of PCV at 8 months of age or greater in a 3 + 0 cluster.

The unit of inference will be the individual child. That is, we wish to estimate whether the risk of VT carriage in a child who lives in a geographic area using a 1 + 1 schedule is not greater than the given threshold when compared to a child in an area receiving the 3 + 0 schedule. The primary outcome will be presented as the proportion (prevalence) of children with clinical pneumonia and NP carriage of VT pneumococci. The contrast between the two groups will be presented as a prevalence ratio (and 95% confidence intervals), with ratios expressed as the prevalence in the 1+ 1 group compared to the prevalence in the 3 + 0 group. To account for cluster-level non-independence, the primary analysis will use generalised estimating equations (GEE) with a log link, binomial family and exchangeable correlation structure. The GEE models will adjust for stratification variables, and age will be included in the model for the endpoint of VT carriage in unimmunised infants aged 6-12 weeks, due to rapid age-related changes in prevalence in the first months of life. Non-inferiority will be established if the upper limit of the confidence interval around the prevalence ratio is ≤1.38.

We have *a priori* interest to examine the following potential effect modifiers: (a) age (2–12 weeks, 13 weeks–9 months, 10–23 months and 24–59 months), (b) inpatient/outpatient status and c) severity (severe pneumonia defined as clinical pneumonia with O_2_ saturation < 92%). The number of cases of each endpoint in the subgroups above may not be sufficient to assess non-inferiority in those strata but may enable the detection of differences in effectiveness between groups. We will examine the strength of evidence for differences between subgroups using interaction tests.

A secondary exploratory measurement and analysis of the primary endpoint will be conducted in year 2 of the trial, in order to detect potential early development of superior impact in the 1 + 1 group. This secondary hypothesis is different to the primary hypothesis of non-inferiority and will use an alpha value of 0.05, as will the final analysis.

The secondary endpoints of community pneumococcal carriage will be measured in years 2 and 4 of the study while carriage in infants aged 6–12 weeks presenting for their first dose of PCV will be measured in year 4. Comparison of the two groups will be presented as a prevalence ratio (and 95% confidence intervals). The same analytical methods as the primary endpoint analysis will be used to account for cluster-level non-independence and the allocation of clusters. We will use the same non-inferiority measurement as the primary endpoint, where non-inferiority will be established if the upper-limit of the confidence interval around the prevalence ratio for VT carriage in the 1 + 1 versus 3 + 0 clusters is ≤1.38.

An exploratory analysis will be performed to measure the association between age-specific contact pattern frequencies and pneumococcal carriage. The association between physical and non-physical contact frequency and age-specific pneumococcal carriage will be analysed using GEE models.

The endpoints monitoring potentially different disease risk (incidence of clinical and radiological pneumonia, IPD and mortality) will be evaluated in semi-blinded safety analyses presented to the DMC at 1, 2, 3, and 4 years after initiation of the interventions. These will not be formal analyses and will not influence the risk of false positive results. These analyses will be concealed from the investigators. The DMC may choose to use the results from the exploratory measurement of the primary endpoint in Year 2 to initiate further safety analyses and measurement of the incidence of clinical pneumonia associated with NP carriage of VT pneumococci. The DMC may also use the results of the community carriage survey in year 2 to inform considerations of safety. These analyses will be concealed from the investigators.

The degree of evidence sufficient to justify early stopping of the trial may be complicated by different accrual rates and potential local epidemics in different clusters. Thus, within and between cluster information will be evaluated for the DMC’s deliberations. The disadvantages of stopping a trial early, such as lack of credibility, imprecision and bias, will be accentuated in cluster-randomised studies.

### Interim analyses {21b}

There are no planned formal interim analyses.

### Methods for additional analyses (e.g. subgroup analyses) {20b}

An exploratory analysis to estimate the magnitude of the herd impact comparing endpoints in unvaccinated children across the two trial groups. This analysis may be limited by the low proportion of children that are unvaccinated.

### Analysis methods to handle non-adherence or missing data {20c}

The clusters will be analysed according to their random allocation. A per-protocol analysis will be performed including clusters that achieve pre-set thresholds for coverage of completed vaccine schedules. An intention to treat analysis will also be performed including all clusters. Sensitivity analyses will include a cluster-level term for the proportion of participants complying with the allocated schedule.

### Plans to give access to the full protocol, participant level-data and statistical code {31c}

The protocol is available on request. The data generated will be suitable for sharing in an anonymised format. The data will be in CDISC ODM format that is an internationally recognised standard. Data will not be deposited into a central repository but held securely by MRCG. The trial is registered with the International Society Clinical Trial Registry Network (ISCTRN) to maximise its visibility to other interested parties. Summary data will be provided through ISCTRN at the end of the study. Data sharing will follow MRC policy [51]. Access to the complete datasets will need to be approved by application to the MRCG Unit’s Archives department who will then forward it to the Scientific Coordinating Committee (SCC) of the Unit. All requests for the dataset will be reviewed by the SCC and also by The Gambia Government/MRC Joint Ethics Committee (GG/MRCG JEC) to establish that the request is in order to perform scientifically appropriate analysis. The datasets collected within the trial will be available to other users once all relevant trial-related publications in scientific journals have been accepted. Prior to this point, requests will be considered on a case by case basis.

- For practical reasons this time period may be indicative and might need to be revised if delays occur. Different periods may be applied to different datasets, e.g. to take into account the complexity of cleaning and documentation.
- Timing will depend on the trial’s collection patterns.
- In relation to timing, the terms could, for instance, be expressed as follows: ‘6-months after the end of the current grant period’, ‘12-months after new data collection to allow for data cleaning and documentation’, or ‘3-months following the first publication of findings based on the data’.

Statistical code will be available on request.

## Oversight and monitoring

### Composition of the coordinating centre and trial steering committee {5d}

The trial management group meets every week and includes the Principle Investigator, Trial Epidemiologist, Trial Coordinator, Data Manager, and Higher Laboratory Scientific Officer. The Trial Steering Committee (TSC) is composed of a Chairperson, an expert clinician, an expert trialist, an expert laboratory scientist, the national EPI Programme Manager, a member of the URR RHD, two community representatives, and a Sponsor representative. The TSC meets one to two months after every DMC meeting. The TSC sets targets for recruitment, data collection, and protocol compliance. All trial-related complaints are reviewed by the TSC. A statistical analysis plan will be approved by the TSC. The TSC considers new information relevant to the trial, including reports from the DMC and the results of other studies that may have a direct bearing on the future conduct of the trial.

### Composition of the data monitoring committee, its role and reporting structure {21a}

The DMC is independent of the Sponsor and composed of a Chairperson, expert clinician, expert statistician, and an independent statistician. The role of the DMC is described in its Charter (see Supplementary material) and is to protect and serve trial participants and to assist and advise the PI so as to protect the validity and credibility of the trial. The DMC monitors the safety of the trial, reviews its progress and accruing data, makes recommendations to the TSC whether the trial should continue, be terminated, or modified, and determines if interim analyses should be undertaken. The DMC also considers data quality, recruitment, compliance with the protocol, sample size assumptions, data emerging from other related studies, requests for interim trial data, and the final data and its interpretation. Materials and discussion during meetings are confidential. The DMC meets every 4–6 months.

### Adverse event reporting and harms {22}

Adverse events are defined according to the ICH Harmonised Guideline for GCP E6(R2). Due to the established safety record of PCV13 the trial does not record solicited events of reactogenicity. The trial uses an electronic vaccine record system to record unsolicited events of reactogenicity when reported by caregivers within 7 days of a dose of PCV. Serious adverse events (SAEs) are tabulated in reports every 3 months. Severe adverse drug reactions (SADR) and serious, severe unexpected serious adverse reactions (SUSAR) are reported to the Sponsor within 24 h. The Sponsor reports SADRs and SUSARS to the Gambia Medicines Control Agency (GMCA), while PI reports these events to the Gambia Government/MRCG Joint Ethics Committee (GG/MRCG JEC), LSHTM Ethics Committee (LSHTM EC), TSC, and DMC.

Follow-up and resolution of SAEs is recorded in electronic reports. The Sponsor, TSC and DMC are informed of SAEs at each meeting. The MRCG Clinical Trials Department Coordinator reports SAEs to the national Regulatory Authority (RA) Gambia Medicine Control Agency (GMCA). The PI reports SAEs to the TSC, DMC, and MoH. SADRs and SUSARs are reported to the GG/MRC JEC within 5 working days. Deaths unrelated to the intervention are reported to the GG/MRC JEC at the next meeting. Information on unanticipated changes that may increase the risk to participants or may affect adversely the safety of the participants or the conduct of the trial or that could alter the EC’s approval to continue the trial will be reported to the Sponsor, GG/MRC JEC, LSHTM EC, GMCA, TSC, and DMC in writing within 2 working days.

### Frequency and plans for auditing trial conduct {23}

The GG/MRCG JEC may audit the conduct of the trial at any time, independent of the investigators and Sponsor. The trial management group meets weekly to review progress in recruitment, clinical endpoint surveillance, and data quality. The trial management group meets monthly to review standardised indicators of quality assurance of all study procedures and complaints. The TSC meets one to two months after every DMC meeting, which occurs approximately every 4–6 months. The TSC sets targets for recruitment, data collection, and protocol compliance. All trial-related complaints are reviewed by the TSC. The trial statistical analysis plan will be submitted for approval by the TSC. The TSC considers new information relevant to the trial, including reports from the DMC and the results of other studies that may have a direct bearing on the future conduct of the trial. Annual reports are submitted to the GG/MRCG JEC and LSHTM EC which document progress in recruitment, SAEs, and protocol deviations and violations.

### Plans for communicating important protocol amendments to relevant parties (e.g. trial participants, ethical committees) {25}

Protocol amendments are reported to the Sponsor, trial Monitors, SCC, GG/MRCG JEC, LSHTM EC, GMCA, DMC, TSC, and clinical trial registry (ISRCTN). Deviations from the protocol are fully documented using a non-adherence/compliance report form.

### Dissemination plans {31a}

The investigators intend to publish the results of the trial in peer-reviewed scientific journals. All trial publications will follow MRC guidelines for Open Access publishing. The results of the study will be disseminated to the parents/guardians of each participant and the communities in the trial area. The findings of the trial will be presented to the Central Stakeholders Committee that includes representatives of the MoH EPI and other regional and central health authorities. The results of the trial will be presented to the WHO EPI department and its Strategic Advisory Group of Experts on Immunisation.

## Discussion

The start of recruitment in the trial was delayed 17 months in 2018/2019 during negotiation of the formal agreement with the MoH regarding the collaborative approach to the trial. Recruitment began on 22 August 2019. Enrolment in the trial was suspended for 4 months in 2020, from 26 March until 30 June, due to restrictions associated with the COVID-19 pandemic. Recruitment in the 1 + 1 clusters resumed from 1 July and was then again suspended on 5 August due to pandemic related restrictions. During these periods of suspension, participants who were already enrolled continued to receive the trial allocated schedules as recorded on infant health cards. The DMC, TSC, and Sponsor, deemed there was less risk to participants if MoH EPI staff followed the schedule indicated on the infant welfare card rather than ask EPI staff to administer PCV in a schedule contrary to that recorded on the infant welfare card. Attendance at immunisation clinics in the trial area was reduced for several months during this time but immunisation of infants was largely caught up when attendance returned to normal 3–4 months after the initial introduction of pandemic related restrictions. Clinical surveillance at health facilities was scaled back on 26 March to the two main hospitals and nursing staff were withdrawn from nine outlying clinics. Collection of NP swabs was suspended from 26 March–7 September and surveillance continued only for safety endpoints of hospitalisation and invasive pneumococcal disease. Disease surveillance was expanded site by site between 28 May and 3 August to then include all health facilities.

Infants resident in 1+ 1 villages who were not enrolled during the COVID-19 interruptions and who may have received the standard 3 + 0 schedule will constitute ‘cross-over’ between the two groups. Cross-over in 1 + 1 clusters will reduce any difference in effect between the two groups with a potential bias towards a null result. A formal evaluation in September 2020, of pandemic-related cross-over, found 1.6% of infants enrolled in the 1+ 1 group were cross-overs during this period while the proportion of all cross-overs in the 1+ 1 group since the beginning of the trial was 4.4%, primarily related to decline of consent. We project that in year 4 of the trial total cross over in the 1 + 1 group will be 3–4%. Formal statistical simulations indicated that such cross-over will have a negligible impact on study power, statistical coverage, or bias.

## Trial status

The current protocol version is 4.0, dated 16 November 2020. From the beginning of recruitment on 22 August 2019 until 17 December 2021, including the periods of pandemic-related suspension, we have recruited 18,882 infants at EPI clinics. Recruitment is ongoing, with the 4-year intervention period projected to end in October 2023.

## Supplementary Information

**Additional file 1:** Supplementary material

## Abbreviations

BHDSS: Basse Health and Demographic Surveillance System
CRF: Case Report Form
CRR: Central River Region
DMC: Data Monitoring Committee
EC: Ethics Committee
eCRF: Electronic Case Report Form
EPI: Expanded Programme on Immunisation
FWHDSS: Fuladu West Health and Demographic Surveillance System
GCP: Good Clinical Practice
GG: Gambia Government
GG/MRCG JEC: Gambia Government/Medical Research Council Gambia Joint Ethics Committee
GMCA: The Gambia Medicines Control Agency
ICH: International Conference on Harmonization
IMCI: Integrated Management of Childhood Illness
IPD: Invasive pneumococcal disease
ISRCTN: International Standard Clinical Trial Registry Network
LSHTM: London School of Hygiene & Tropical Medicine
LSHTM EC: London School of Hygiene & Tropical Medicine Ethics Committee
MCRI: Murdoch Children’s Research Institute, Melbourne
MoH: Ministry of Health
MRCG: Medical Research Council Unit The Gambia
NP: Nasopharyngeal
NVT: Non-vaccine type
PCV: Pneumococcal Conjugate Vaccine
PI: Principal Investigator
PSP: Pneumococcal Surveillance Project
PVS: Pneumococcal Vaccine Schedules study
RA: Regulatory Authority
RCH: Reproductive Child Health
SAE: Serious adverse event
SCC: Scientific Coordinating Committee
SGUL: St Georges University of London
STGGB: Skim milk tryptone glucose glycerol broth
SUSAR: Serious Unexpected Serious Adverse Reaction
TSC: Trial Steering Committee
URR: Upper River Region
VT: Vaccine-type
WHO: World Health Organization
